# Simultaneous development of sarcoidosis and cutaneous vasculitis in a patient with refractory Crohn’s disease during infliximab therapy

**DOI:** 10.1186/s12890-016-0193-5

**Published:** 2016-02-11

**Authors:** Tadahisa Numakura, Tsutomu Tamada, Masayuki Nara, Soshi Muramatsu, Koji Murakami, Toshiaki Kikuchi, Makoto Kobayashi, Miho Muroi, Tatsuma Okazaki, Sho Takagi, Yoshinobu Eishi, Masakazu Ichinose

**Affiliations:** Department of Respiratory Medicine, Tohoku University Graduate School of Medicine, 1–1 Seiryo-machi, Aoba-ku, Sendai, 980–8574 Japan; Clinical Research, Innovation and Education Center, Tohoku University Hospital, Sendai, Japan; Department of Respiratory Medicine and Infectious Diseases, Niigata University Graduate School of Medical and Dental Sciences, Niigata, Japan; Takagi Clinic, Sendai, Japan; Department of Human Pathology, Tokyo Medical and Dental University, Tokyo, Japan

**Keywords:** Paradoxical inflammation, Anti-tumor necrosis factor-alpha, *Propionibacterium acnes*

## Abstract

**Background:**

Paradoxical inflammations during anti-TNF-α therapy are defined as adverse effects such as psoriasiform skin lesions, uveitis and sarcoidosis-like granulomas induced by immune reactions, not by infectious agents. Here, we report a very rare case of the simultaneous development of sarcoidosis and cutaneous vasculitis in a patient with refractory Crohn’s disease during infliximab therapy and both of which resolved spontaneously without the cessation of infliximab.

**Case presentation:**

In September 2000, 23-year old Japanese male was diagnosed with Crohn’s disease. Prednisolone in combination with mesalazine was introduced at first and succeeded for almost one year. In June 2002, since his gastrointestinal symptoms relapsed and were refractory, infliximab (IFX) therapy 5 mg/kg was introduced. In February 2011, because he had repeated arthralgia almost every intravenous IFX administration, IFX was increased to 10 mg/kg under the diagnosis of a secondary failure of IFX. In December 2012, he complained of slight dry cough and an itchy eruption on both lower limbs, and he was referred to our hospital due to the appearance of bilateral hilar lymphadenopathy on chest X-ray examination. Chest computed tomogram revealed bilateral hilar lymphadenopathy and fine reticulonodular shadows on the bilateral upper lungs. Serum calcium, angiotensin-converting enzyme and soluble interleukin 2 receptor levels were not elevated, but the titer of antinuclear antibody was considerably elevated. *Mycobacterium* infection was carefully excluded. Trans-bronchial lung biopsy showed non-caseating epithelioid cell granulomas compatible with sarcoidosis. The skin biopsy of the right limb was diagnosed as leukocytoclastic vasculitis. The patient was diagnosed as having a series of paradoxical inflammations during anti-TNF-α therapy. Since his paradoxical inflammations were not severe and opportunistic infections were excluded, IFX was cautiously continued for refractory Crohn’s disease. Nine months later, not only his intrathoracic lesions but also his cutaneous lesions had spontaneously resolved.

**Conclusion:**

Physicians caring for patients with anti-TNF-α therapy should know that, based on a careful exclusion of infectious agents and thoughtful assessment of the patient’s possible risks and benefits, paradoxical inflammations can be resolved without the cessation of anti-TNF-α therapy.

## Background

Tumor necrosis factor-alpha (TNF-α) is known to be one of the key cytokines for granuloma formation in both sarcoidosis and inflammatory bowel diseases. Infliximab, which is a humanized chimeric monoclonal antibody against TNF-α, is indicated for refractory Crohn’s disease, and several reports have described its effectiveness for refractory sarcoidosis. As the use of anti-TNF-α therapy has been increasing, various paradoxical inflammations such as cutaneous vasculitis, drug induced lupus and sarcoidosis have been reported in recent years [1]. We describe a patient of refractory Crohn’s disease who developed sarcoidosis and cutaneous vasculitis simultaneously during anti-TNF-α antibody, infliximab, therapy.

## Case presentation

**Fig. 1:**
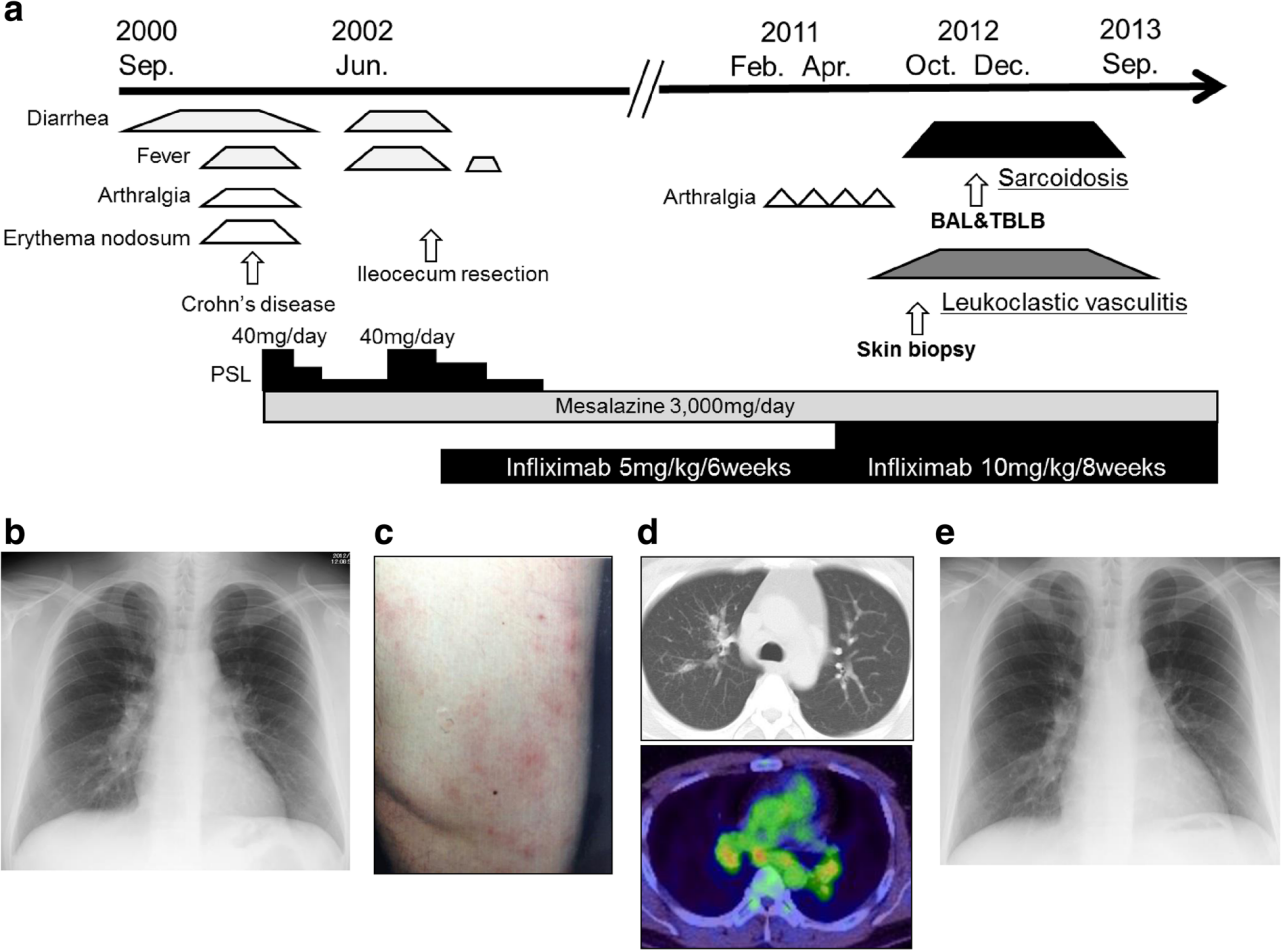
**a** A chart delineating the clinical course of this case. After infliximab was increased to 10 mg/kg for every 8 weeks, both sarcoidosis and leukocytoclastic vasculitis developed. **b** A chest X ray on admission revealed bilateral hilar lymphadenopathy. **c** Scattered eruption on the right thigh on admission. **d** A Chest CT scan on admission revealed bilateral hilar lymphadenopathy and fine reticulo-nodular shadows in the peri-bronchovascular region of the bilateral upper lobes (upper panel) and positron emission tomography (FDG-PET) scan indicated increased FDG uptakes in the bilateral hilar lymph nodes (lower panel). **e** Nine months later, the bilateral hilar lymphadenopathy and the upper lobe reticulo-nodular shadows had spontaneously resolved

In September 2000, 23-year old Japanese male developed melena, fever, arthralgia and erythema nodosum (Fig. 1a). After close examination, he was diagnosed with Crohn’s disease. Prednisolone (PSL) (40 mg/day) in combination with mesalazine (3000 mg/day) was introduced at first, and then PSL was subsequently tapered to the maintenance dose (5 mg/day). These induction therapies succeeded in achieving partial remission for almost one year. In June 2002, his gastrointestinal symptoms relapsed, but the increase of PSL to 40 mg/day failed to achieve remission. After infliximab (IFX) therapy (intravenous IFX 5 mg/kg every 6 weeks) was introduced, his disease gradually became well controlled. In February 2011, he had repeated arthralgia almost every 6 weeks after intravenous IFX. In the following April, IFX was increased to 10 mg/kg for every 8 weeks under the diagnosis of a secondary failure of IFX at the local clinic.

**Fig. 2:**
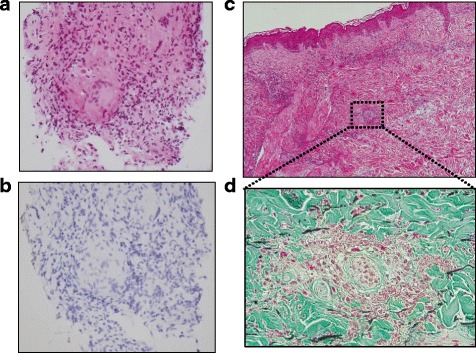
**a** Trans-bronchial lung biopsy (TBLB) revealed non-caseating epithelioid cell granuloma with Langhans giant cells, which were compatible with sarcoidosis (HE staining, ×400). **b** Anti *Propionibacterium acnes* antibody staining was negative within this specimen. **c** Skin biopsy from the right thigh shown in Fig. 1d revealed leukocytoclastic vasculitis in the superficial layer of the sub-dermal portion. (HE staining, ×100). **d** A high-power field of Fig. 2c revealed remarkable infiltration of neutrophils around small capillaries and leukocytoclasis. (Elastica-Masson staining, ×400)

In October 2012, he complained of slight dry cough and an itchy eruption on both lower limbs, and he was referred to our hospital due to the appearance of bilateral hilar lymphadenopathy on chest X-ray examination (Fig. 1b). On admission, his vital signs were normal and an erythema on both lower limbs was present (Fig. 1c). Laboratory findings were almost within normal limits. Serum calcium, angiotensin-converting enzyme (ACE) and soluble interleukin 2 receptor (sIL-2R) levels were not elevated, but the titer of antinuclear antibody (ANA) was considerably elevated to ×1280. A purified derivative skin test (PPD) was negative (3 mm × 3 mm). *Mycobacterium tuberculosis* (Mtb) infection was carefully excluded because the result of QuantiFERON-TB 3-G (QFT3-G) was negative where Mtb antigen value was 0.11 IU/ml, nil value 0.12 IU/ml and mitogen (positive control) value over 10 IU/ml, and both repeated sputum cultures and polymerase chain reaction (PCR) of sputum samples as well as that of biopsy material taken at bronchoscopy were all negative for Mtb. Chest computed tomogram (CT) revealed mediastinal and bilateral hilar lymphadenopathy, and fine reticulonodular shadows in broncho-vascular bundles on the bilateral upper lung fields (Fig. 1d, upper panel). Positron emission tomography (FDG-PET) scan indicated abnormally increased FDG uptakes in the mediastinal and bilateral hilar lymph nodes (Fig. 1d, lower panel), suggesting the possibility of sarcoidosis or malignant lymphoma. Broncho-alveolar lavage fluid (BALF) showed an increase in the ratio of lymphocyte counts (44 %), but the ratio of CD4/8 was not elevated (1.1). The culture in BALF was negative for any pathogens including Mtb. Trans-bronchial lung biopsy (TBLB) showed several non-caseating epithelioid cell granulomas including Langhans giant cells in the interstitium of the peripheral alveolar legions (Fig. 2a). These findings were compatible with sarcoidosis. Because *Propionibacterium acnes* (*P. acnes*) is the only bacteria that has been cultured from sarcoid-granuloma [2], we investigated immunohistochemical staining using anti *P. acnes* antibody, but immunoreactivity was negative in the TBLB specimens (Fig. 2b). The skin biopsy specimen of the right limb was diagnosed as leukocytoclastic vasculitis (Fig. 2c, d), which is the most common type of vasculitis that occurs during anti-TNF-α therapy. Based on the simultaneous development of sarcoidosis and cutaneous vasculitis, the patient was diagnosed as having a series of paradoxical inflammations during anti-TNF-α therapy. Since opportunistic infections were carefully excluded, his paradoxical inflammations were not severe and he wanted to continue anti-TNF-α therapy for his refractory Crohn’s disease, IFX (10 mg/kg for every 8 weeks) was continued cautiously. Nine months later, not only his intrathoracic lesions but also his cutaneous lesions had spontaneously improved without any opportunistic infections in the lungs while under IFX treatment (Fig. 1e).

## Discussion

TNF-α is one of the key cytokines in the formation of non-caseating epithelioid cell granulomas in both sarcoidosis [3, 4] and Crohn’s disease [5]. IFX, anti-TNF-α antibody, is widely used for refractory Crohn’s disease and there is increasing evidence that it is effective for refractory sarcoidosis [6]. Recently, it has been reported that anti-TNF-α therapy induces so-called paradoxical inflammations, including anti-nuclear antibody production, drug induced lupus, vasculitis and sarcoid-like granulomatous disease [1, 7–10]. Although the mechanism responsible for the development of sarcoidosis during anti-TNF-α therapy is not well understood, the simultaneous development of sarcoidosis and cutaneous vasculitis in the present case may suggest an underlying pathogenesis for the paradoxical inflammations. To the best of our knowledge, this is the first case that simultaneously developed sarcoidosis and cutaneous vasculitis during IFX therapy for refractory Crohn’s disease.

### Sarcoidosis during anti TNF-α therapy

In 2010, Ramos-Casals and colleagues reported that there had been 38 cases of sarcoidosis or “sarcoid like” granulomatous disease during anti-TNF-α therapy out of 1370 adult patients of systemic autoimmune diseases under the use of biological agents [9, 10]. Etanercept (ETN), a humanized TNF-α2 receptor, was used in 75 % of the cases, but IFX was used in only 25 % of the cases. Anti-TNF-α therapy was withheld in most of these cases because of the possibility of respiratory infection or severe symptoms of sarcoidosis, and in some cases systemic corticosteroid and/or anti tuberculosis therapy were tried. In only a few cases, as well as in our case, anti-TNF therapy was continued carefully after opportunistic infections were excluded, because the original diseases were severe and could not be controlled without anti-TNF therapy. Both symptoms and abnormal findings caused by sarcoidosis spontaneously resolved in those cases. Since it can be difficult to determine whether anti-TNF-α therapy should be continued or not, a course of treatment must be based on a careful assessment of the patient’s possible risks and benefits.

### Differences between IFX and ETN in host immunity

There are some structural and pharmacological differences between ETN and IFX [11]. It is speculated that because the effect of ETN is weaker and shorter in terms of its TNF-α neutralization capacity than that of IFX, reactivated TNF-α is viable and more likely to induce paradoxical reactions. IFX is a chimeric monoclonal antibody of mouse Fab and human IgG1 Fc. IFX binds both monomer and trimer forms of soluble TNF-α and form a stable complex. IFX also forms a stable complex with transmembrane forms of TNF-α on the surface of activated inflammatory cells. This leads to ADCC (antibody-dependent cell-mediated cytotoxity), CDC (complement-dependent cytotoxity) and reverse signaling, strongly inducing the apoptosis of inflammatory cells. Thus, IFX is effective for the treatment of Crohn’s disease. On the other hand, IFX compromises the patient’s immunity, resulting in the reactivation of intracellular latent infection by *P. acnes* or Mtb. *P. acnes* is the only bacteria that has been cultured from sarcoid-granuloma [2], and Mtb catalase-peroxidase (mKatG) was reported to be a pathogenic antigen in sarcoidosis [12]. Although the *P.acnes* antibody staining was negative in our TBLB specimen, this may not exclude the possibility of reactivation of intracellular latent infection, because a TBLB specimen is too small for *P.acnes* antibody staining and it is difficult to detect the dormant phase of *P. acnes*. It is important to closely investigate the possibility of above mentioned microorganisms to determine whether anti-TNF-α therapy should be continued or not.

### Possible mechanisms of simultaneous development of both sarcoidosis and leukocytoclastic vasculitis as paradoxical inflammations during anti-TNF-α therapy

It is known that some patients develop a so-called secondary failure, in which a symptom of the original disease relapses and infusion reaction may appear in the course of IFX treatment. A neutralizing auto-antibody against IFX (anti-IFX Ab) may be deeply related to the pathogenesis of this secondary failure [13]. According to a previous report [14], the speculated mechanisms of anti-IFX Ab production are as follows. First, IFX induces apoptosis in pathogenic macrophages, resulting in a reduction of TNF-α production and granuloma formation in the intestinal submucosa. Next, inadequately activated T lymphocytes recognize the apoptotic debris including auto-antigens. Then, the further activated Th1 and Th2 system induces production of auto-antibodies such as ANA, anti-double strand DNA (dsDNA) antibody and also anti-IFX Ab [1]. Because IFX is a mouse/human chimeric anti TNF-α antibody, IFX is more likely to trigger anti-IFX Ab production than ETN, a synthesized anti TNF-α receptor [13]. It has also been reported that the appearance of anti-IFX Ab is closely related to the occurrence of ANA or anti-DNA-antibody [15]. Finally, anti-IFX Ab and ANA production is likely to cause sarcoid-like granulomatous disease and leukocytoclastic vasculitis, respectively. To date, 113 cases of vasculitis during anti-TNF therapies have been reported [16]. The most common clinical manifestation is cutaneous vasculitis, which is pathologically compatible with leukocytoclastic vasculitis. It has been suggested that when immune complex formations such as TNFα/anti-TNFα Ab or autoantigen/ANA are deposited in small capillaries, complement cascades are activated and type III hypersensitivity reaction may occur. Additionally, immune dysregulation or a cytokine imbalance may underlie the development of vasculitis in patients during anti-TNF therapies.

### Inflammatory bowel disease and sarcoidosis

In a review addressing thoracic manifestations of inflammatory bowel disease [17], it was reported that there have been 53 cases of inflammatory bowel disease coexisting with sarcoidosis; underlying ties between the two entities were also suggested. Later in 2012, Colin et al. mentioned that, based on recent genome wide analyses, a susceptibility locus for sarcoidosis is shared by Crohn’s disease [18]. It is also difficult in clinical settings to differentiate whether the appearance of sarcoid-like granulomas during anti-TNF-α therapy be true paradoxical inflammations or intrathoracic manifestation of Crohn’s disease or co-existence of Crohn’s disease and sarcoidosis.

## Conclusion

In summary, we report a very rare case of the simultaneous development of sarcoidosis and cutaneous vasculitis in a patient with refractory Crohn’s disease during IFX therapy and both of which resolved spontaneously without the cessation of IFX. Physicians caring for patients with anti-TNF-α therapy should determine whether anti-TNF-α therapy be continued or not, based on a careful exclusion of infectious agents and thoughtful assessment of the patient’s possible risks and benefits.

### Ethics

This case report has been granted an exemption from requiring ethics approval in Tohoku University Graduate School of Medicine, but all the process has been done in accordance with the Declaration of Helsinki.

## Consent

Written, informed consent was obtained from the patient for publication of this case report and all accompanying images. A copy of the written consent is available for review by the editor of this journal.

## Abbreviations

IFX: infliximab
FDG-PET: positron emission tomography with 2-deoxy-2-[fluorine-18]fluoro - D-glucose
ETN: etanercept
PCR: polymerase chain reaction
PSL: prednisolone
Mtb: *mycobacterium tuberculosis*
TNF-α: tumor necrosis factor-alpha
